# Relationship between eating attitudes, depression, and insight in schizophrenic patients with and without type 2 diabetes mellitus: a comparative study in Guangdong, China

**DOI:** 10.3389/fpsyt.2024.1477953

**Published:** 2024-10-03

**Authors:** Wenqing Zhou, Weiye Cao, Wen Wang, Gang Zeng, Rongyu Liang, Cuixia Liu, Xiaodong Chen, Weicheng Lin, Xiaolei Shi, Huarong Zhou, Yun Gao, Wanhua Chen, Lingxian Xiao

**Affiliations:** ^1^ Adult Psychiatry Department, The Affiliated Brain Hospital, Guangzhou Medical University, Guangzhou, China; ^2^ Key Laboratory of Neurogenetics and Channelopathies of Guangdong Province and the Ministry of Education of China, Guangzhou Medical University, Guangzhou, China; ^3^ School of Nursing, Guangzhou Medical University, Guangzhou, China; ^4^ Geriatric Neuroscience Center, The Affiliated Brain Hospital, Guangzhou Medical University, Guangzhou, China; ^5^ Department of Geriatric Psychiatry, The Affiliated Brain Hospital, Guangzhou Medical University, Guangzhou, China; ^6^ Chronic Psychiatry Department, The Affiliated Brain Hospital, Guangzhou Medical University, Guangzhou, China; ^7^ Department of Nursing, The Affiliated Brain Hospital, Guangzhou Medical University, Guangzhou, China

**Keywords:** schizophrenia, type 2 diabetes mellitus, eating attitudes, depression, insight

## Abstract

**Background:**

Schizophrenia, a severe mental disorder, is often complicated by Type 2 Diabetes Mellitus (T2DM), which can further impact patients’ psychological health. This study investigated the differences in eating attitudes, depression, and insight between schizophrenic patients with and without comorbid T2DM and explored the correlations among these factors to provide empirical support for clinical interventions.

**Methods:**

This case-control study was conducted in Guangdong Province, China. From December 2022 to May 2023, a total of 300 hospitalized patients with schizophrenia (92 with comorbid T2DM and 208 without T2DM) were recruited. Data were collected using the Personal Information Form, Eating Attitudes Test (EAT-26), Hamilton Depression Scale (HAMD), and Insight and Treatment Attitudes Questionnaire (ITAQ). Statistical analyses, including t-tests, ANOVA, and multiple linear regression, were performed to examine differences and predictive factors of eating attitudes among patients. This study was approved by the Ethics Committee of the Affiliated Brain Hospital of Guangzhou Medical University (approval number: 2020028), and written informed consent was obtained from all participants.

**Results:**

Patients with schizophrenia and comorbid T2DM exhibited significantly higher risks of eating disorders (EAT-26: 12.54 ± 9.77 vs. 9.07 ± 7.90, *P*=0.003), more severe depression (HAMD: 14.71 ± 7.36 vs. 11.80 ± 6.04, *P*=0.001), and poorer insight (ITAQ: 10.46 ± 6.01 vs. 12.16 ± 6.09, *P*=0.025) compared to those without T2DM. Regression analysis revealed that gender, weekly exercise frequency, depression, and insight were significant predictors of eating attitudes among patients with T2DM. For patients without T2DM, weekly exercise frequency, smoking status, and insight were significant predictors.

**Conclusion:**

Schizophrenic patients with comorbid T2DM are facing increasing risks related to eating attitudes, depression, and insight which highlight the need for targeted interventions. Regular psychological assessment and tailored support strategies might improve their mental health and quality of life. Future research should focus on longitudinal studies to clarify causal relationships and develop more effective interventions.

## 1 Introduction

Schizophrenia is a severe mental disorder affecting approximately 24 million people worldwide (1). It significantly impacts cognitive, emotional, and behavioral functions, leading to a decline in social functioning (2). Schizophrenia often manifests in young adults and tends to be chronic and recurrent; imposing a substantial burden on patients, their families, and society (3). Additionally, the life expectancy of individuals with schizophrenia is 10-20 years shorter than that of the general population, and their mortality rate is twice as high (4). The increased mortality rate is partly due to non-natural causes such as suicide; however, it is largely attributed to the higher mortality associated with comorbid conditions (5).

In recent years, the high prevalence of type 2 diabetes mellitus (T2DM) among patients with schizophrenia has drawn widespread attention. T2DM is a chronic metabolic disorder characterized by hyperglycemia, usually caused by inadequate insulin secretion or action (6). Among patients with schizophrenia, the prevalence of T2DM may be as high as 20% (7), and the risk of developing T2DM is at least twice that of the general population (8). The increased prevalence of T2DM in patients with schizophrenia is attributed to multiple factors. Traditional factors include smoking, poor dietary habits, and lack of physical activity. Specific risk factors for schizophrenia include low socioeconomic status, cognitive impairment, and metabolic side effects caused by antipsychotic medications (9). Furthermore, there might be shared genetic factors between schizophrenia and T2DM (10).

The presence of diabetes not only increases the physical burden on patients with schizophrenia but also exacerbates their mental health issues. Firstly, patients with T2DM, due to the need for strict management of diet and medication, often experience higher psychological stress, which may worsen their depressive symptoms (11). Chronic inflammatory response and oxidative stress caused by diabetes can lead to neurotransmitter imbalances and neuronal dysfunction, thereby increasing the risk of depression in patients with schizophrenia (12). Additionally, diabetes may further affect patients’ cognitive function and emotional regulation by influencing the levels of neurotrophic factors, such as brain-derived neurotrophic factor (BDNF). Generally, a reduction in BDNF levels is associated with cognitive impairment and depressive symptoms (13). However, some studies have shown that serum BDNF levels in patients with schizophrenia and comorbid T2DM are significantly higher than in those without diabetes (14), suggesting that the mechanism of BDNF changes may differ due to the presence of diabetes and requires further research. Recent studies have also revealed the inflammatory mechanisms of the interaction between T2DM and schizophrenia. Chronic low-grade inflammation related to diabetes may interact with neuroinflammatory responses in schizophrenia, potentially leading to further deterioration of cognitive function and emotional state (15, 16). Some studies have indicated that elevated levels of C-reactive protein (CRP) and interleukin-6 (IL-6) in diabetic patients are closely related to the worsening of symptoms and increased depression levels in patients with schizophrenia (17). Therefore, comorbid diabetes not only affects depression and insight but may also adversely impact other psychological and behavioral issues in patients with schizophrenia through multiple mechanisms.

Among these psychological and behavioral issues, eating disorders are an important area that has received widespread attention in patients with schizophrenia. Patients with schizophrenia are inherently more likely to develop eating disorders due to food-related delusions, hallucinations, and side effects of antipsychotic medications (18–20). Furthermore, proper nutrition is crucial for the effectiveness of antipsychotic treatment, and disordered eating behaviors, such as binge eating or excessive dieting, in patients with schizophrenia can easily affect the efficacy of medications, leading to the recurrence of psychotic symptoms (21). In this context, comorbid diabetes complicates the issue further. Diabetic patients require strict dietary management, and this pressure can lead to excessive focus on weight and diet, increasing the risk of binge eating or poor eating control (22, 23).

Although previous studies have found that diabetes in the general population can lead to depression and eating disorders (11, 24–26), researches on patients with schizophrenia and comorbid diabetes have primarily focused on poor prognosis, increased mortality, and mechanistic studies (9, 27–29). There is currently a lack of systematic studies exploring how diabetes specifically affects psychological factors such as eating attitudes, depression, and insight in patients with schizophrenia. Therefore, this study aims to compare the differences in these psychological factors between patients with schizophrenia with and without comorbid T2DM, further revealing the interactions between these factors and providing empirical support for future clinical intervention strategies.

## 2 Materials and methods

### 2.1 Subjects

This study employed purposive sampling to recruit 300 inpatients with schizophrenia from a tertiary psychiatric hospital in Guangdong Province between December 2022 and might 2023. Group A consisted of 92 patients with comorbid type 2 diabetes mellitus and schizophrenia, while Group B included 208 patients with schizophrenia only. Inclusion criteria were: (1) age over 18 years; (2) diagnosis of schizophrenia according to ICD-11 criteria; (3) hospitalization of ≥6 months; (4) stable condition at the time of data collection, defined as scores of ≤3 on items P1 (delusions), P2 (conceptual disorganization), P3 (hallucinations), and P6 (suspiciousness/persecution) of the Positive and Negative Syndrome Scale (PANSS), with normal perception and communication abilities; (5) willingness to sign the informed consent form. Exclusion criteria: (1) other mental diseases such as dementia, substance dependence, bipolar disorder were excluded; (2) combined severe physical diseases, such as cardiovascular, hepatic, or renal diseases; (3) unable to complete clinical data collection. This study was approved by the Ethics Committee of the Affiliated Brain Hospital of Guangzhou Medical University (approval number: 2020028), and written informed consent was obtained from all participants.

### 2.2 Questionnaire

#### 2.2.1 General information

The clinical data including gender, age, Body Mass Index (BMI), education background, marital status, kid status, smoking, and frequency of exercise (weekly) was collected by using a self-designed demographic questionnaire.

#### 2.2.2 Eating attitudes test-26

The Eating Attitude Test (EAT-26), developed by Garner et al., operates as a self-evaluation tool for assessing an individual’s eating attitudes (30). It encapsulates three specific dimensions: dietary habits, associated with the avoidance of fatty foods and inclination towards slimness; bulimia and food preoccupations, correlated with continued consideration of food and bulimic tendencies; and oral control, relating to the self-regulation of eating behaviors and external stressors concerning weight gain. This psychometric evaluation includes 25 items, each scored on a four-point scale: “always” is accorded three points, “usually” merits two points, “often” ascribes one point, whilst “sometimes”, “rarely”, and “never” receive zero points each. The 26th item, however, is an exception—it is scored inversely. The final score constitutes a summation of points from all factors, producing a possible range between zero and 78. In the EAT-26 framework, a score exceeding 20 is indicative of a significant risk for the development of an eating disorder (31). In this study, the EAT-26 demonstrated good internal consistency with a Cronbach’s α coefficient of 0.869.

#### 2.2.3 Hamilton rating scale for depression

Hamilton introduced the Hamilton Depression Rating Scale, a scale comprising 24 targeted queries designed to categorize the intensity of depression in subjects. This scale represents a precise depiction of disease severity, exhibiting strong reliability and validity within a potential scoring range of 0 to 96. The HAMD primarily implements a five-point grading system, where 0 means “none,” 1 signifies “mild,” 2 connotes “moderate,” 3 implies “severe,” and 4 represents the pinnacle of depression. A minor segment employs a three-point grading strategy, assigning 0-2 points (0 as “none,” 1 as “doubtful or mild,” and 2 as blatant symptoms). The sum of these individual HAMD scores yields the total score. A total of fewer than 8 points is classified as normal, scores between 8 and 20 suggest potential depression, 21 to 35 points constitute diagnosed depression, and scores exceeding 35 depict profound depression respectively (32). In this study, the HAMD showed good internal consistency with a Cronbach’s α coefficient of 0.822.

#### 2.2.4 Insight and treatment attitudes questionnaire

The Insight and Treatment Attitudes Questionnaire (33) (ITAQ) is utilized to assess insight by administering an assessment tool comprising 11 items. These items are designed to gauge individuals’ recognition of mental disorders and their attitudes towards medication, hospitalization, and follow-up care. Each question in the questionnaire is rated on a 0 to 2 scale, with 0 denoting no insight, 1 indicating partial insight, and 2 signifying good insight. The questionnaire has a total score range of 0 to 22. Participants scoring above 18 are categorized as possessing high levels of insight, while those with scores below 6 are deemed to lack insight. A higher ITAQ score indicates a greater depth of insight in patients. In this study, the ITAQ indicated good internal consistency with a Cronbach’s α coefficient of 0.921.

### 2.3 Sample size

To ensure the accuracy and reliability of the results, the sample size typically needs to be 5-10 times the number of independent variables. Specifically, the sample size calculation formula could be expressed as: sample size = number of independent variables × (5 -10). This study used 11 variables. Therefore, the preliminary estimate required 110 cases. Considering a 15% rate of invalid data, the final estimate needed 127 samples. This study included 300 subjects. All subjects met the inclusion and exclusion criteria.

### 2.4 Data collection

A team of five experienced nurses conducted interviews with the subjects and distributed questionnaires. All nurses underwent standardized training before data collection to ensure consistency and accuracy in the questionnaire collection process. Each nurse communicated with the subjects through face-to-face interviews and cross-checked the information with the patient’s primary nurse after the questionnaire was completed to ensure the reliability and validity of the data. A total of 315 questionnaires were distributed, and 300 were completed, resulting in a completion rate of 95.2%. Among the 15 incomplete questionnaires, 4 patients withdrew after consenting but before the survey, 9 patients chose to withdraw during the survey, and 2 patients refused to submit the questionnaire after completing it due to concerns about information leakage.

### 2.5 Statistical analysis

SPSS 25.0 software was used for data analysis. Comparative analysis was performed between patients with comorbid diabetes and schizophrenia and those with schizophrenia alone. Categorical data were analyzed using the chi-squared test. Continuous data were analyzed using the t-test and one-way analysis of variance (ANOVA). Multiple linear regression analysis was also performed. The EAT-26 score was used as the dependent variable and statistically significant factors were used as independent variables.

## 3 Results

### 3.1 Characteristics of patients with schizophrenia with and without comorbid T2DM

Regarding sociodemographic variables, there were no statistical differences between schizophrenic patients with and without T2DM, except for gender (*P*=0.046). The proportion of male patients without T2DM (65.38%) was higher than that of male patients with T2DM (53.26%). Other variables, such as age, BMI, education level, marital status, kid status, smoking status, and exercise frequency, showed no significant differences between the two groups (*P*>0.05) (**Table 1**). These results suggested that the two groups of patients are similar in most demographic characteristics, and gender differences might be a factor to consider in further analysis.

**Table 1:** Characteristics of Patients with Schizophrenia with and without Comorbid T2DM.

Characteristic	T2DM *N* (%)	Without T2DM *N* (%)	*X^2^*	*P-value*
Gender			3.97	0.046*
Male	49(53.26)	136(65.38)		
Female	43(46.74)	72(34.62)		
Age (years old)			0.45	0.798
<45	8(8.70)	23(11.06)		
45-59	35(38.04)	74(35.58)		
≥60	49(53.26)	111(53.37)		
BMI			2.40	0.663
Underweight	6(6.52)	11(5.29)		
Standard	43(46.74)	98(47.12)		
Overweight	22(23.91)	61(29.33)		
Mild obesity	15(16.30)	31(14.90)		
Moderate obesity	6(6.52)	7(3.37)		
Education			1.90	0.594
Uneducated and primary school	10(10.87)	20(9.62)		
Middle school	31(33.70)	85(40.87)		
High school	37(40.22)	69(33.17)		
University and above	14(15.22)	34(16.35)		
Marital status			2.27	0.518
Single	56(60.87)	135(64.90)		
Married	18(19.57)	40(19.23)		
Divorced	15(16.30)	31(14.90)		
Widowed	3(3.26)	2(0.96)		
Kid status			0.08	0.994
None	66(71.74)	152(73.08)		
1	21(22.83)	46(22.12)		
2	3(3.26)	6(2.88)		
≥3	2(2.17)	4(1.92)		
Smoking status			0.04	0.851
Never	19(20.65)	41(19.71)		
Yes, now smoke	73(79.35)	167(80.29)		
Frequency of exercise (Weeks)			2.91	0.406
0	10(10.87)	28(13.46)		
1~2	27(29.35)	47(22.60)		
3~4	11(11.96)	37(17.79)		
>4	44(47.83)	96(46.15)		

*P < 0.05.

### 3.2 EAT-26, HAMD, and ITAQ scores of patients with schizophrenia with and without comorbid T2DM

The patients were divided into two groups based on whether they had T2DM: the group with T2DM and the group without T2DM. The two groups were compared in terms of EAT-26, HAMD, and ITAQ scores of patients. The total EAT-26 score for patients with schizophrenia with comorbid T2DM was 12.54 ± 9.77, which was significantly higher than that of patients with schizophrenia only (9.07 ± 7.90, *P*=0.003). This suggested that T2DM may have an important impact on the eating behaviors of these patients. The average HAMD score for patients with schizophrenia with comorbid T2DM was 14.71 ± 7.36, which was also significantly higher than that of patients with schizophrenia only (11.80 ± 6.04, *P*=0.001), indicating that T2DM might negatively affect the mental health of these patients by increasing depressive symptoms. In addition, the average ITAQ scores for the two groups were 10.46 ± 6.01 and 12.16 ± 6.09, respectively, with a statistically significant difference (*P*=0.025), indicating that patients with comorbid T2DM may have poorer insight (**Table 2**).

**Table 2:** Comparison of EAT-26, HAMD, and ITAQ Scores between Patients with Schizophrenia with and without Comorbid T2DM.

Variables	T2DM Mean ± SD	without T2DM Mean ± SD	*t*	*P-value*
EAT-26	12.54 ± 9.77	9.07 ± 7.90	3.01	0.003**
HAMD	14.71 ± 7.36	11.80 ± 6.04	3.32	0.001**
ITAQ	10.46 ± 6.01	12.16 ± 6.09	-2.25	0.025*

*P < 0.05, **P < 0.01.

### 3.3 Comparison of EAT-26 scores according to different characteristics in patients with schizophrenia with and without comorbid T2DM

The two groups were compared in terms of EAT-26 scores according to different characteristics. **Table 3** showed the comparison results of EAT-26 scores under different characteristics. Among patients with schizophrenia and comorbid T2DM, female patients (14.93 ± 10.11) and patients with lower weekly exercise frequency (0 times per week: 18.60 ± 8.04) had significantly higher EAT-26 scores than male patients (10.45 ± 9.06) and those with higher exercise frequency (1-2 times per week: 14.30 ± 10.21; 3-4 times per week: 13.36 ± 8.32; more than 4 times per week: 9.89 ± 9.57) (*P*< 0.05). Similarly, among patients with schizophrenia only, female patients (10.68 ± 9.26), those with lower education levels (no education and primary school: 14.60 ± 9.71), lower weekly exercise frequency (0 times per week: 14.36 ± 10.27), and those who smoked (13.48 ± 9.94) had higher EAT-26 scores (*P*< 0.05). These results indicated that gender, exercise frequency (weekly), education level, and smoking status might be important factors influencing eating attitudes.

**Table 3:** Comparison of EAT-26 scores according to different characteristics in patients with schizophrenia with and without comorbid T2DM.

Characteristic	T2DM Mean ± SD	*P-value^a,b^*	without T2DM Mean ± SD	*P-value^a,b^*
Gender				
Male	10.45 ± 9.06	0.027^a^	8.21 ± 6.97	0.050^a^
Female	14.93 ± 10.11		10.68 ± 9.26	
Age (years old)				
<45	14.13 ± 9.57	0.890^b^	8.70 ± 5.76	0.920^b^
45-59	12.51 ± 9.90		9.35 ± 7.77	
≥60	12.31 ± 9.90		8.96 ± 8.41	
BMI				
Underweight	16.33 ± 10.61	0.458^b^	8.64 ± 6.92	0.404^b^
Standard	12.81 ± 9.99		9.10 ± 8.26	
Overweight	13.23 ± 9.90		9.02 ± 7.90	
Mild obesity	11.80 ± 9.99		7.97 ± 6.45	
Moderate obesity	6.16 ± 5.08		14.57 ± 9.85	
Education				
Uneducated and primary school	14.10 ± 11.66	0.392^b^	14.60 ± 9.71	0.010^b^
Middle school	13.84 ± 10.13		8.19 ± 7.37	
High school	12.51 ± 9.75		8.90 ± 8.02	
University and above	8.64 ± 7.24		8.35 ± 6.75	
Marital status				
Single	13.29 ± 10.00	0.300^b^	9.78 ± 8.13	0.261^b^
Married	13.61 ± 11.24		7.60 ± 7.46	
Divorced	10.20 ± 6.96		7.61 ± 6.93	
Widowed	4.00 ± 2.65		14.14 ± 10.00	
Kid status				
None	13.11 ± 9.90	0.654^b^	9.67 ± 8.08	0.121^b^
1	10.24 ± 9.20		8.20 ± 7.68	
2	15.00 ± 10.15		4.50 ± 2.88	
≥3	14.50 ± 13.26		3.00 ± 1.41	
Smoking status				
Never	8.95 ± 8.40	0.072^a^	6.49 ± 5.38	0.019^a^
Yes, now smoke	13.48 ± 9.94		9.70 ± 8.30	
Frequency of exercise (Weeks)				
0	18.60 ± 8.04	0.042^b^	14.36 ± 10.27	<0.001^b^
1~2	14.30 ± 10.21		9.96 ± 7.92	
3~4	13.36 ± 8.32		10.05 ± 7.20	
>4	9.89 ± 9.57		6.71 ± 6.43	

SD, standard deviation; a, t-test; b, ANOVA.

### 3.4 Predictive factors of EAT-26 scores in patients with schizophrenia with and without comorbid T2DM

Using EAT-26 as a dependent variable, the following variables of patients in Group A were used as independent variables: gender, frequency of exercise, HAMD scores and ITAQ scores. As for Group B, using EAT-26 as a dependent variable, the following variables of patients in Group A were used as independent variables: gender, frequency of exercise, education, smoking status, HAMD scores and ITAQ scores. The multiple linear regression model was constructed, shown in **Table 4**. **Table 4** presented the results of the multiple linear regression analysis of the predictive factors for EAT-26 scores. Among patients with schizophrenia and comorbid T2DM, the model explained 40.3% of the variance (adjusted *R*²=0.43, *F*=16.38, *P*<0.001). EAT-26 scores were significantly correlated with gender (*β*=3.59, *P*= 0.030), exercise frequency (*β*=-2.32, *P*=0.002), depression (HAMD scores, *β*=0.26, *P*=0.036), and insight (ITAQ scores, *β*=-0.72, *P*<0.001). Exercise frequency and insight were negatively correlated with EAT-26 scores, indicating that more frequent exercise and better insight might be associated with a lower risk of eating disorders. For patients with schizophrenia only, the model explained 18.0% of the variance (adjusted *R*²=0.18, *F*=8.59, *P*<0.001). EAT-26 scores were significantly correlated with weekly exercise frequency (*β*=-1.99, *P*<0.001), smoking status (*β*=3.16, *P*=0.018), and insight (*β*=-0.27, *P*=0.003).

**Table 4:** Predictive Factors of EAT-26 Scores in Patients with Schizophrenia with and without Comorbid T2DM.

Variables	*B* (95%*CI*)	*SE*	*β*	*t*	*P*
Constant (T2DM)	17.89	3.76		4.76	<0.001
Gender	3.59	1.63	0.18	2.21	0.030
Frequency of exercise	-2.32	0.73	-0.26	-3.20	0.002
depression	0.26	0.12	0.19	2.13	0.036
Insight	-0.72	0.14	-0.44	-4.99	<0.001
*R^2 =^ 0.43, Adjusted R^2 =^ 0.40, F=16.38, P<0.001*					
Constant (without T2DM)	11.90	3.36		3.54	<0.001
Gender	1.01	1.12	0.06	0.91	0.366
Frequency of exercise	-1.99	0.45	-0.28	-4.42	<0.001
Education	-0.83	0.58	-0.09	-1.44	0.152
Smoking status	3.16	1.33	0.16	2.38	0.018
depression	0.12	0.09	0.09	1.39	0.165
Insight	-0.27	0.09	-0.21	-3.06	0.003
*R^2 ^=*0.20, *Adjusted R^2 ^=*0.18, *F*=8.59, *P<0.001*					

*B*, unstandardized coefficients; *β*, standardized regression coefficient. *CI*, confidence interval; *R* ^2^, coefficient of determination.

## 4 Discussions

To our knowledge, this is one of the few studies investigating whether T2DM affects psychological factors in patients with schizophrenia. The main findings of this study are: 1) patients with schizophrenia and comorbid T2DM had a higher risk of eating disorders, more severe depressive symptoms, and poorer insight compared to patients with schizophrenia only; 2) multiple regression analysis showed that in patients with schizophrenia and comorbid T2DM, gender, exercise frequency, depression, and insight were predictive factors for eating attitudes.

Our study found that patients with schizophrenia and comorbid type 2 diabetes mellitus (T2DM) had significantly higher EAT-26 scores than those with schizophrenia only (12.54 ± 9.77 vs. 9.07 ± 7.90, *P*=0.003), indicating a higher risk of eating disorders in patients with schizophrenia and comorbid T2DM. A study using the EAT-26 as an assessment tool also found similar results, where adolescents with type 1 diabetes mellitus were more likely to exhibit severe disordered eating behaviors than their non-diabetic peers (22). The reasons for this might relate to diabetes management, self-esteem, and body image. Gagnon et al. found that self-management of both type 1 and type 2 diabetes mellitus was associated with dietary care, which could lead to rigid attitudes toward eating, thereby increasing the risk of over-concern with weight and eating, binge eating, and dysfunctional behaviors (23). Over-concern with dietary rules might inhibit patients’ physiological sensations and promote psychological and physiological deprivation, making them more prone to eating disorder behaviors. In addition to causing eating disorders, diabetes could lead to frustration and affect self-esteem and confidence in managing diabetes. This frustration often arises because healthcare providers might be disappointed with the patients’ poor adherence to diabetes management (34). Moreover, self-esteem and body image could decline following the diagnosis and treatment of diabetes mellitus (35).

Furthermore, our study found that patients with schizophrenia with comorbid T2DM had significantly higher HAMD scores compared to those with schizophrenia only (14.71 ± 7.36 vs. 11.80 ± 6.04, *P*=0.001). This result suggests that patients with comorbid T2DM experience more severe depression. The reasons might be related to the higher psychological stress typically faced by patients with schizophrenia, and the additional diagnosis of diabetes and strict self-management requirements may further exacerbate this stress, increasing the severity of depressive symptoms (36). Previous studies have suggested that there may be a bidirectional relationship between depression and T2DM (11, 37). On one hand, depression may lead to a lack of physical exercise, antidepressant treatment, and weight gain, all of which could increase the risk of developing type 2 diabetes mellitus (11). On the other hand, the psychological stress and diabetes-related distress (including strict self-management and concerns about disease complications) caused by T2DM could exacerbate depressive symptoms (38). Our study also found that depression was an important predictor of eating attitudes (*β*=0.19, *P*=0.036). This result suggests that higher levels of depression in patients with schizophrenia with comorbid T2DM might be associated with unhealthier eating attitudes. Previous research has shown that depressive symptoms can affect an individual’s emotional regulation ability, making them more prone to unhealthy eating behaviors such as binge eating or over-concern with diet (39). Depression may lead to difficulties in dietary control and self-management, further exacerbating disordered eating behaviors. For patients with schizophrenia and comorbid T2DM, the higher the level of depression, the worse their eating attitudes may be, which could form a vicious cycle. Therefore, clinical interventions should focus not only on diabetes management and schizophrenia treatment but also on depression management, improving patients’ emotional state through psychological therapies such as cognitive behavioral therapy (CBT), and promoting healthier eating behaviors and overall health.

Our study also indicated that patients with schizophrenia and comorbid T2DM had significantly lower insight scores compared to those with schizophrenia only (ITAQ scores: 10.46 ± 6.01 vs. 12.16 ± 6.09, *P*=0.025), suggesting poorer insight and treatment attitude levels in patients with comorbid T2DM. Similar to the findings of Zhu et al., chronic schizophrenia patients with comorbid diabetes mellitus often lack insight, have poor self-control, and low treatment adherence (40). Poor insight may mean that patients have difficulty fully understanding the overall impact of both their mental illness and diabetes, especially regarding the importance of diabetes management and treatment adherence. Under these circumstances, patients may be more likely to neglect the complexities of disease management and the long-term health risks. Shen et al. found that in patients with schizophrenia during antipsychotic treatment, metabolic factors such as baseline T4 levels and waist-to-hip ratio (WHR) were significantly associated with weight gain, further highlighting that poor knowledge of metabolic health and disease management in these patients could lead to adverse health outcomes (41). Additionally, our study found that insight was a significant negative predictor of eating attitudes (*β*=-0.44, *P*<0.001), indicating that poorer insight might lead to unhealthier eating attitudes. This is particularly evident in patients with schizophrenia with comorbid T2DM. Patients with low insight often lack awareness of the importance of changing healthy behaviors and may be more inclined to maintain unhealthy eating behaviors or lack the motivation for dietary control. Şenay and Yücel reported a significant association between low insight levels and a higher risk of eating disorders, which is consistent with our findings (42). Furthermore, patients with low insight may be more reliant on pharmacological treatment (such as atypical antipsychotic medications), which may lead to increased appetite and weight gain, thereby worsening the risk of diabetes and eating disorders (Zhu et al., (40)). Therefore, improving insight might be a key strategy for reducing eating disorders and enhancing overall health management in patients with schizophrenia and comorbid T2DM. Future research and clinical practice should focus on enhancing patients’ insight through education and psychological support to promote more proactive health behaviors and better treatment adherence.

The analysis of factors influencing eating attitudes in this study indicated a correlation between gender and eating attitudes in patients with schizophrenia and comorbid T2DM. This finding is consistent with previous research indicating that women with diabetes mellitus are at a higher risk of developing eating disorders compared to men, as women tend to be more concerned with being thin and their body weight (25). Another explanation is that high EAT-26 scores in men are related to a history of fitness and previous obesity, and EAT-26 scores in young boys are highly correlated with BMI and decrease with age, suggesting that concern about body shape and weight might decrease as men age (43). In this study, the frequency of exercise was associated with improved eating attitudes in patients with schizophrenia with comorbid T2DM. Previous research has shown that higher levels of physical activity could increase motivation for healthier eating behaviors (44). Other studies further explain that physical activity could influence food rewards, with increased habitual physical activity levels being associated with reduced rewards for high-fat or high-energy foods and a preference for low-fat/low-energy foods (45). Additionally, our study found that smoking status was significantly related to eating attitudes in patients with schizophrenia only, but not in those with comorbid T2DM. Smoking may affect eating behaviors through nicotine’s suppressive effect on brain appetite control and its ability to relieve anxiety and stress. Patients may cope with negative emotions by binge eating, while smoking could alleviate such emotional stress, thereby altering their eating attitudes (46).

This study has some limitations. Firstly, the cross-sectional design limits the ability to infer causality among eating attitudes, depression, and insight. Since data were collected at a single time point, we cannot determine the causal pathways among these psychological factors, which may affect the interpretation of the results in clinical applications. Future studies should consider adopting a longitudinal design to track changes in these psychological factors and clarify their causal interactions. Secondly, the sample in this study was drawn from a single tertiary psychiatric hospital, which may limit the external validity of the results and affect their generalizability to different healthcare settings or regions. Future research should include multi-center and multi-regional samples to improve the generalizability and applicability of the study findings.

## 5 Conclusion

This study found that patients with schizophrenia and comorbid T2DM faced higher risks in terms of eating attitudes, depression, and insight. Among these patients, women and those with low levels of physical activity were particularly prone to developing eating disorders. The study suggests that the presence of T2DM might exacerbate issues related to eating attitudes and depressive symptoms in patients with schizophrenia. This could be attributed to the additional disease burden brought about by diabetes, which further impacts their mental health. Improving insight may help enhance patients’ eating attitudes and overall health status. Future studies should further explore the interactions among these psychological factors and develop more effective interventions to improve the health and quality of life of patients with schizophrenia and comorbid T2DM.

## Data Availability

The raw data supporting the conclusions of this article will be made available by the authors, without undue reservation.
